# Which head element is more effective for cement augmentation of TFNA? Helical blade versus lag screw

**DOI:** 10.1186/s12891-023-06671-9

**Published:** 2023-07-03

**Authors:** Sadaki Mitsuzawa, Takeharu Nakamata, Shogo Mitamura, Tadashi Yasuda, Shuichi Matsuda

**Affiliations:** 1 Department of Orthopaedic Surgery, Kobe City Medical Center General Hospital, 2-1-1 Minatojima-Minamimachi, Chuo-Ku, Kobe, 650-0047 Japan; 2 Department of Orthopaedic Surgery, Rakuwakai Otowa Hospital, Kyoto, Japan; 3 Department of Orthopaedic Surgery, Kyoto University Graduate School of Medicine, Kyoto, Japan

**Keywords:** Proximal femur fracture, Trochanteric fixation nail advanced (TFNA), Cement augmentation, Cement distribution, Helical blade, Lag screw, Computed tomography (CT)

## Abstract

**Background:**

Early fixation and rehabilitation is the gold standard treatment for intertrochanteric femur fractures. Cement augmentation through perforated head elements has been developed to avoid postoperative complications such as cut-out or cut-through. The purpose of this study was to compare two head elements in terms of cement distribution using computed tomography (CT) and to examine their initial fixation and clinical outcomes.

**Methods:**

Elderly patients who had intertrochanteric fractures were treated with a trochanteric fixation nail advanced (TFNA) helical blade (Blade group) or a TFNA lag screw (Screw group). In both groups, 4.2 mL of cement was injected under an image intensifier (1.8 mL of cement was directed cranially and 0.8 mL each caudally, anteriorly, and posteriorly). Patient demographics and clinical outcome were investigated post-operatively. Cement distribution from the center of the head element was evaluated with CT. Maximum penetration depth (MPD) were measured in the coronal and sagittal planes. On each axial plane, the cross-sectional areas in the cranial, caudal, anterior and posterior directions were calculated. The sum of cross-sectional areas (successive 36 slices) was defined as the volume of the head element.

**Results:**

The Blade group included 14 patients, and the Screw group included 15 patients. In the Blade group, MPD in the anterior and caudal direction was significantly greater than that in the posterior direction (*p* < 0.01). In the Screw group, volume in the cranial and posterior direction was significantly greater than that in the Blade group (*p* = 0.03). Subsequently, the total volume in the Screw group was significantly larger than that in the Blade group (*p* < 0.01). No significant correlation was detected between bone mineral density, T score, young adult mean, and total cement volume. Change in radiographic parameters and clinical outcome such as Parker score and visual analog scale were similar in both groups. No patients suffered from cut-out / cut through or non-union.

**Conclusions:**

The position of cement distribution through the lag screw is different from that through the helical blade, and the total volume of the head element is significantly larger in the lag screw. Both groups had similarly effective results in terms of mechanical stability after surgery, postoperative pain and early phase of rehabilitation.

**Trial registration:**

Current Controlled Trials ISRCTN45341843, 24/12/2022, Retrospectively registered.

## Background

Alongside the increase in the aging population, the number of intertrochanteric femur fractures continues to increase, which has led to a high rate of mortality and morbidity in elderly people. Surgical stabilization, early mobilization, and weight bearing is the gold standard treatment to prevent immobility-related complications. Although surgical fixation using a cephalomedullary nail is the most popular method, the choice of head element depends on the surgeon’s preference. Although a clinical comparison of the new-generation Trochanteric Femoral Nail Advanced (TFNA) helical blade (DePuy Synthes, West Chester, PA, USA) versus the Gamma3 with U-Blade (RC) lag screw (Stryker GmbH, Kiel, Germany) has not yet been reported, numerous biomechanical and clinical studies have compared these two types of head elements [1–4]. The complication rate, such as implant failure, was similar between the two groups, however, cut-out tended to occur in the lag screw and cut-through (medial migration) tend to occur in the helical blade [5–7]. Severe osteoporosis and unstable fracture type as well as inadequate fracture reduction and suboptimal implant position are the risk factors for these complications.

To improve the mechanical stability of head elements in osteoporotic patients, polymethylmethacrylate (PMMA) cement augmentation with perforated head elements for proximal femur fractures was developed and became available in our country in November 2020. Cement augmentation showed promising biomechanical and clinical results due to the bone cement–implant interface, regardless of poor bone stock. There have been no reports of cut-out or cut-through complications with cement augmentation [8, 9]. Because the amount and distribution of injected cement in the femoral head is important for mechanical stability, we previously examined the distribution of cement around the TFNA helical blade in detail using computed tomography (CT) [10]. The TFNA system holds the perforated lag screw as well as the perforated helical blade. In these two types of head elements, the number and position of perforated holes differed. No research has compared the cement distribution and clinical outcome of the TFNA helical blade with cement augmentation versus the TFNA lag screw with cement augmentation.

The purpose of this randomized study was to compare the helical blade and the lag screw in terms of the distribution of injected cement using CT, and to examine its initial fixation of an implant and clinical outcomes. Our null hypothesis is that the cement position and volume might differ, but the stability and clinical results will be similar between the two groups.

## Methods

The study protocol and research were performed in accordance with the Ethics Committee at our institution. This pilot study was designed as a randomized study that compared elderly patients who are undergoing TFNA with cement augmentation. Between 1 November 2020 and 30 April 2021, patients who had intertrochanteric fractures were randomized to two groups. Because of the small number of patients, stratified randomization was performed using age and sex. One group was treated with the TFNA helical blade, and the other was treated with the TFNA lag screw. All patients were blinded to the choice of the head element. Cement augmentation was performed in all patients. Patients 60 years and older who had a closed intertrochanteric fracture (AO Foundation/Orthopaedic Trauma Association (AO/OTA) classification 31A1, A2 and A3) were included in this study. The exclusion criteria were as follows: (1) occult fracture detected by magnetic resonance imaging only; (2) pathological fracture; (3) presence of pre-existing implants; or (4) multiple trauma or additional fracture that would affect the patient’s postoperative rehabilitation.

### Surgical technique and postoperative protocol

**Fig. 1:**
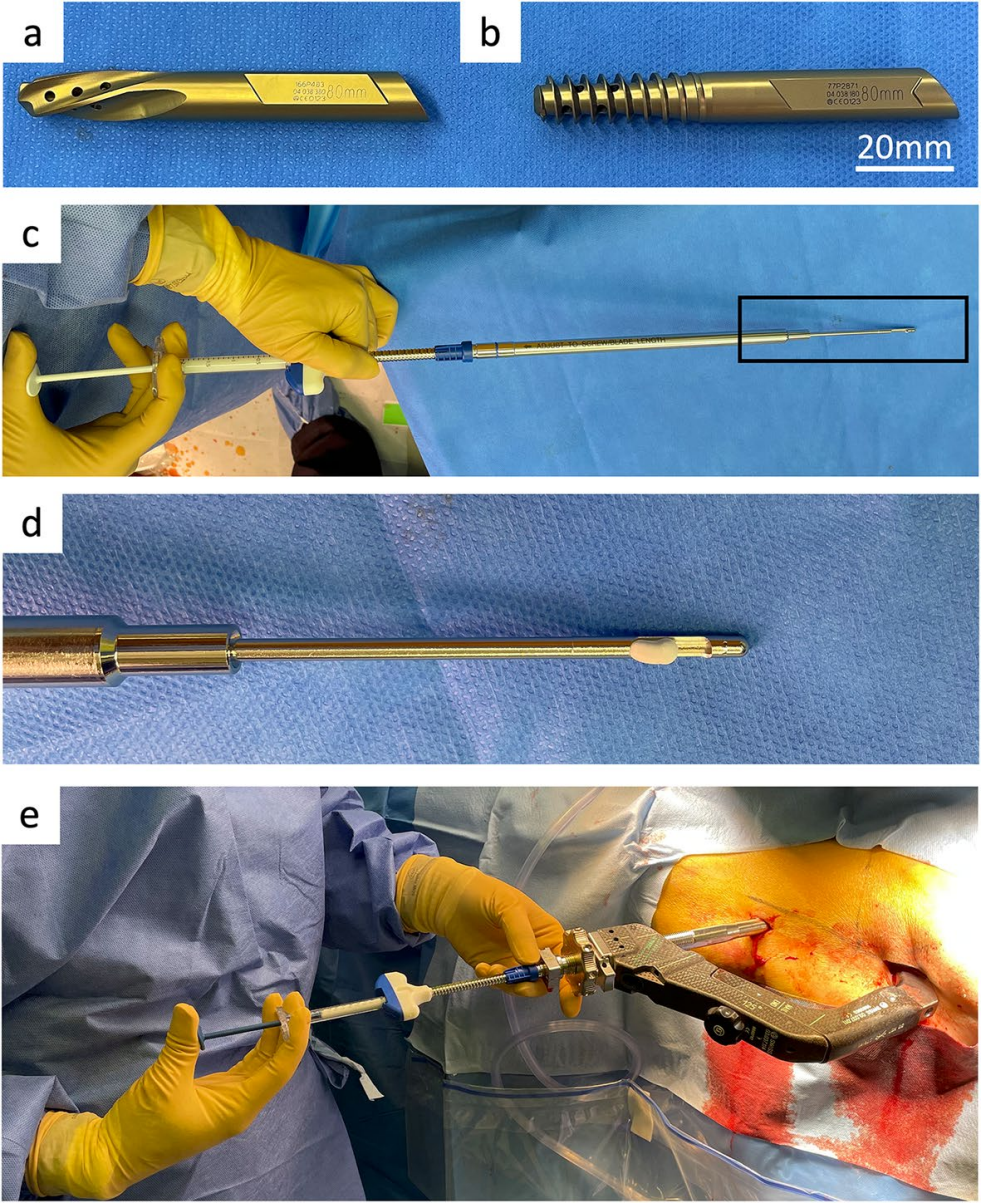
Photograph of the two types of implants and surgical procedure. **A** helical blade. **B** lag screw. **C** cement was loaded up to the tip of side-opening cannula. **D** enlarged image of the black square above; cement reached the window. **E** cement was injected through the device of TFNA

All operations were performed by the first author. The fracture was reduced on a traction table. In cases where the anteromedial cortex of the proximal fragment was wedged into the medullary cavity of the distal fragment, an additional reduction technique was used to achieve the optimal position. After reduction of the fracture, a TFNA (φ10mm, 125°, DePuy Synthes, West Chester, PA, USA) was inserted according to the manufacturer’s instructions. We inserted the helical blade or the lag screw, aiming for the center/center position with 10 mm of tip–apex distance (TAD) at anteroposterior (AP) view, and with 10 mm of TAD at lateral view (Fig. 1A,B). The set screw was loosened by a one-quarter turn to allow postoperative telescoping and fracture compression. Intraoperative compression using a device was not performed in any cases. All patients were treated with cement augmentation. 4.2 mL of PMMA cement (Traumacem V + cement, DePuy Synthes) was injected circumferentially using an image intensifier to monitor AP and lateral views (Fig. 1C-E). By rotating the side-opening cannula, 1.8 mL of cement was directed to the cranial and 0.8 mL each was directed in the other three directions (caudal, anterior, and posterior). The postoperative protocol was identical in both groups; all patients were mobilized under physiotherapeutic supervision with full weight bearing as tolerated starting on the day after surgery.

### Data collection

Patient demographic data included the following: age, gender, body mass index (BMI), diabetes, tobacco use, Charlson comorbidity index, American Society of Anesthesiologists (ASA) status, dual-energy X-ray absorptiometry (DEXA) at the neck (bone mineral density; BMD), T score and young adult mean (YAM), laterality, AO/OTA fracture classification, and pre-fracture ambulatory level (Parker score), follow-up period [11, 12]. The following intraoperative information was recorded: operative time; intraoperative blood loss; presence of intraoperative complications such as tachycardia, rapid decrease in blood pressure, oxygen saturation, or cement leak; and nail length.

### Radiographic parameters

**Fig. 2:**
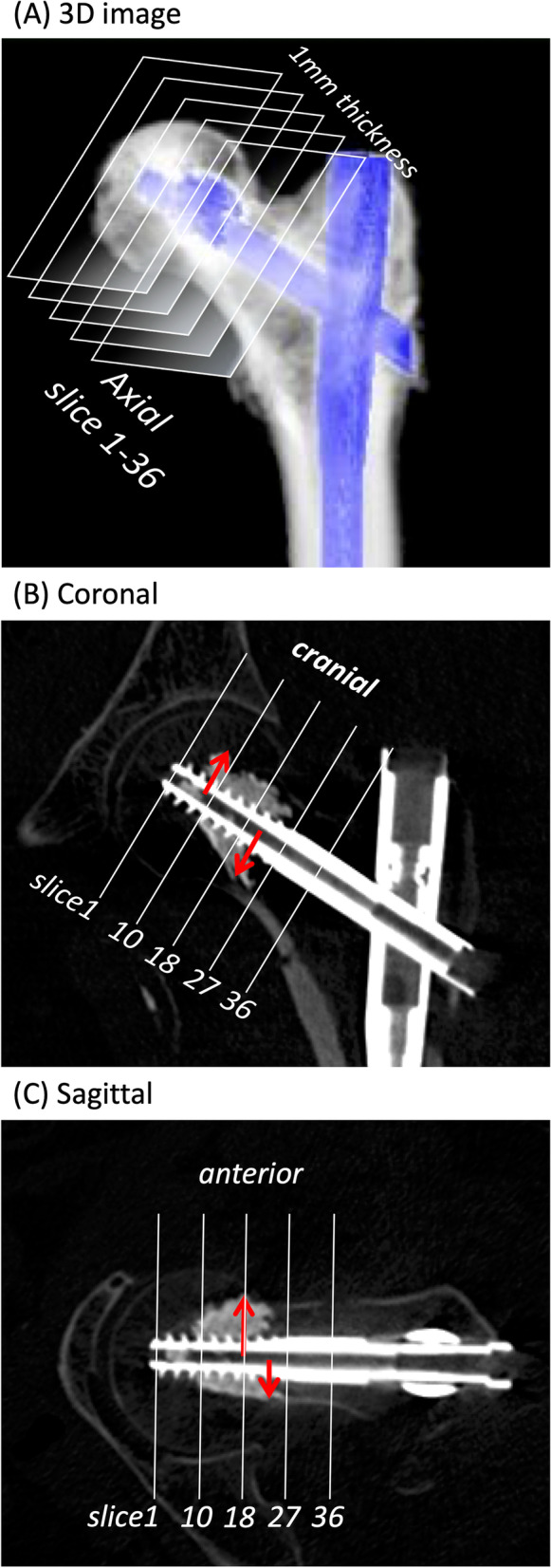
Radiographic parameters using coronal and sagittal CT. **A** 3D image: The head element was cut into round slices in the axial plane: 36 successive 1-mm slices starting at the tip of the head element (slice 1). **B** Coronal plane: where the head element and the inserted nail were placed in the same plane. The maximum penetration depth (MPD) (cranial and caudal) was measured as the distance from the center of the head element to the end of cement distribution (red arrow). **C** Sagittal plane: perpendicular to the coronal plane and parallel to the head element. The MPD (anterior and posterior) was measured (red arrow). The picture above is a lag screw case

**Fig. 3:**
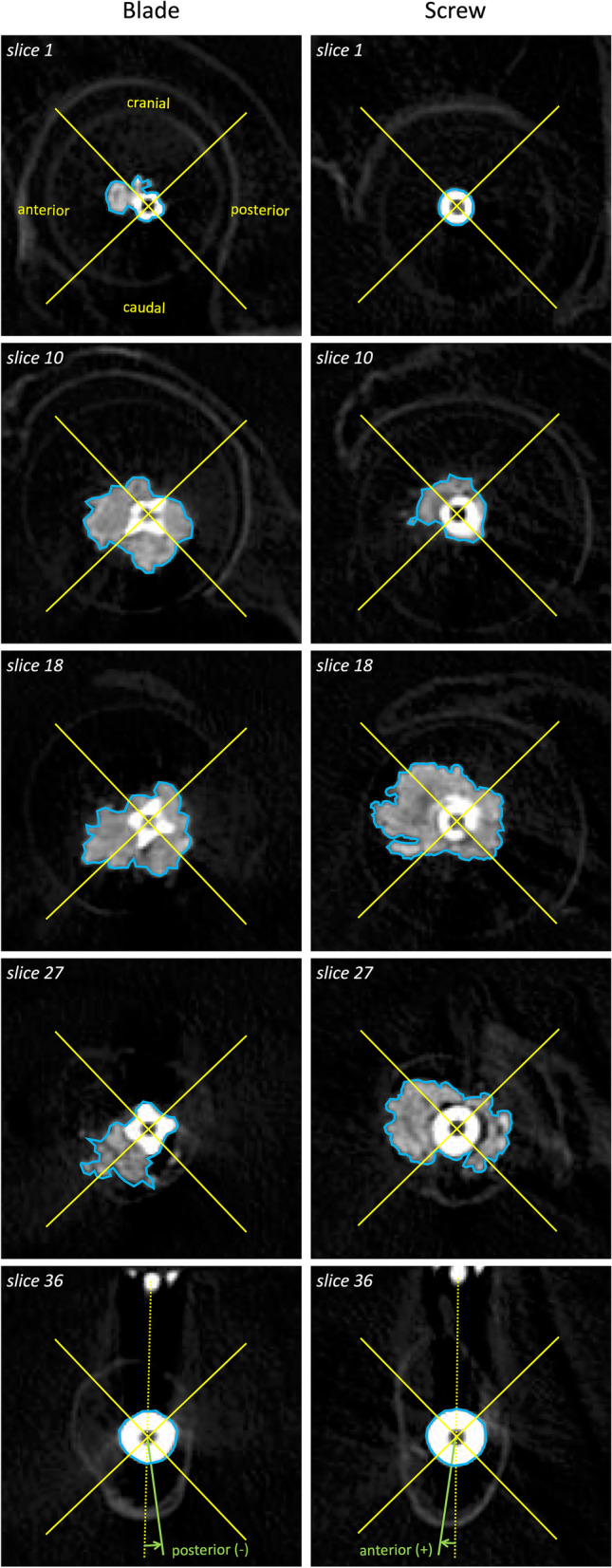
Radiographic parameters using axial CT. In the axial plane, the rotation angle (green arc) was measured on slice 36, which is defined as the angle between the nail–blade line (yellow dotted line) and the blade–medial calcar line (green line). Four directions (cranial, caudal, anterior, and posterior) were defined based on the nail–blade line. On each axial plane through slices 1 to 36, the cross-sectional areas of head elements and dispersed cement (blue polygon shape) were divided into the cranial, caudal, anterior, and posterior parts, and were calculated. In each of the four directions, the sum of cross-sectional areas (total 36 slices) was defined as the volume of the head element. The sum of cement volume in the four directions was defined as the total volume

On the day after surgery, CT evaluation was performed with a window level of 2700 HU and a window width of 5800 HU. This window level and width minimized the artifact of the metal and cement structure when measuring the radiographic parameters below. In the current study, coronal, sagittal, and axial planes were defined as follows: coronal plane, where the head element and inserted nail were placed on the same plane; sagittal plane, which was perpendicular to the coronal plane and parallel to the head element; and axial plane, which was perpendicular to the coronal and sagittal plane. On the axial plane, which had 36 successive 1-mm slices starting at the tip of the head element (slice 1), the head element was cut into round slices (Fig. 2A). We evaluated the position of the head elements within 9 Cleveland zones on the basis of Yam’s criteria [7]. We evaluated the reduction quality on the basis of Yoo’s criteria [4], as follows: “good” (medial calcar cortex of the proximal fragment was positioned in a neutral or medial position compared to the distal fragment in the coronal plane, and the anterior cortex of proximal fragment was positioned in a neutral or anterior position compared to the distal fragment in the sagittal plane), “acceptable” (the reduction met the criteria for a “good” reduction for either view but not both), or “poor” (the reduction met neither criteria). In the coronal plane, TAD (coronal), Parker ratio (coronal), blade end (the amount of head element protrusion from the lateral edge of the nail), and maximum penetration depth (MPD) (cranial and caudal) were measured (Fig. 2B). In the sagittal plane, TAD (sagittal), Parker ratio (sagittal), and MPD (anterior and posterior) were measured, based on previous studies (Fig. 2C) [13, 14]. In the axial plane, the rotation angle, which is defined as the angle between the nail–blade line and blade–medial calcar line on axial slice 36, was measured using the modified method of Yamazaki et al. [15]. Based on the nail–blade line, four directions (cranial, caudal, anterior, and posterior) were defined. On each axial plane through slices 1 to 36, the cross-sectional areas of head elements and dispersed cement were divided into the cranial, caudal, anterior, and posterior parts, and were calculated using ImageJ software (National Institutes of Health, Bethesda, MD, USA) (Fig. 3). In each of the four directions, the sum of cross-sectional areas (total 36 slices) was defined as the volume of the head element. The sum of cement volume in four directions was defined as the total volume. On postoperative day 14, a CT scan was performed, and the difference in the TAD (coronal), TAD (sagittal), blade end, and rotation angle was calculated as delta (Δ).

### Clinical outcome measurement

On postoperative day 14, the Parker score, visual analog scale (VAS) for passive range of motion (ROM), and full-load walking were evaluated. The difference in the Parker score was calculated as delta (Δ). Cut-out / cut-through or non-union were investigated at final follow-up.

### Statistical analysis

Data were presented as the mean ± standard deviation (SD) and analyzed using JMP Pro 15.0 (SAS Institute, Cary, NC, USA). A priori analysis of sample size was calculated with a power of 0.80 / α error of 0.05, using G*power 3.1 (Heinrich-Heine-Universität Düsseldorf, Düsseldorf, Germany), according to the previous study [10]. The Student’s *t*-test was used to compare the demographic data, radiographic parameters, and clinical outcomes of the Blade and Screw groups. Fisher’s exact test was performed to analyze categorical data. Values of *p* < 0.05 were considered to be statistically significant. The Pearson correlation test was applied to analyze the dependencies and correlations between BMD, T score, YAM, and total cement volume.

## Results

During the study period, 29 patients underwent cephalomedullary nailing for intertrochanteric fractures. The Blade group included 14 patients, and the Screw group included 15 patients. All 29 fractures were caused by falls from a standing position. We assessed the patient demographics between the two groups, and they were determined to be well balanced. There was no statistically significant difference between the two groups in terms of BMD, T score, or YAM (Table 1).

**Table 1:** Patients demographics

	Blade group (*n* = 14)	Screw group (*n* = 15)	*p* value
Mean age (years)	82.4 ± 9.6	81.8 ± 14.3	0.89
Male: Female	3: 11	5: 10	0.68
Body mass index (kg/m^2^)	18.5 ± 2.6	19.4 ± 2.3	0.33
Diabetes	4	6	0.70
Tobacco use	2	4	0.65
Charlson comorbidity index	2.7 ± 1.6	3.3 ± 1.2	0.26
ASA status			0.02
1	4	0	
2	2	8	
3	7	5	
4	1	1	
DEXA (neck)			
BMD (g/cm^2^)	0.452 ± 0.192	0.453 ± 0.104	1.00
T score	− 3.6 ± 2.1	− 3.6 ± 1.1	0.99
YAM (%)	57.2 ± 24.3	55.7 ± 12.5	0.84
Right: Left	7: 7	7: 8	
AO/OTA Fracture classification			0.87
31 A1	4	6	
31 A2	8	7	
31 A3	2	2	
Pre-fracture Parker score	5.1 ± 2.5	5.7 ± 1.6	0.47
Follow-up period (months)	17.1 ± 1.9	16.3 ± 1.3	0.22

*ASA* American society of anesthesiologists, *DEXA* Dual-energy x-ray absorptiometry, *BMD* Bone marrow density, *YAM* Young adult mean, *AO/OTA* AO Foundation/Orthopaedic trauma association

**Fig. 4:**
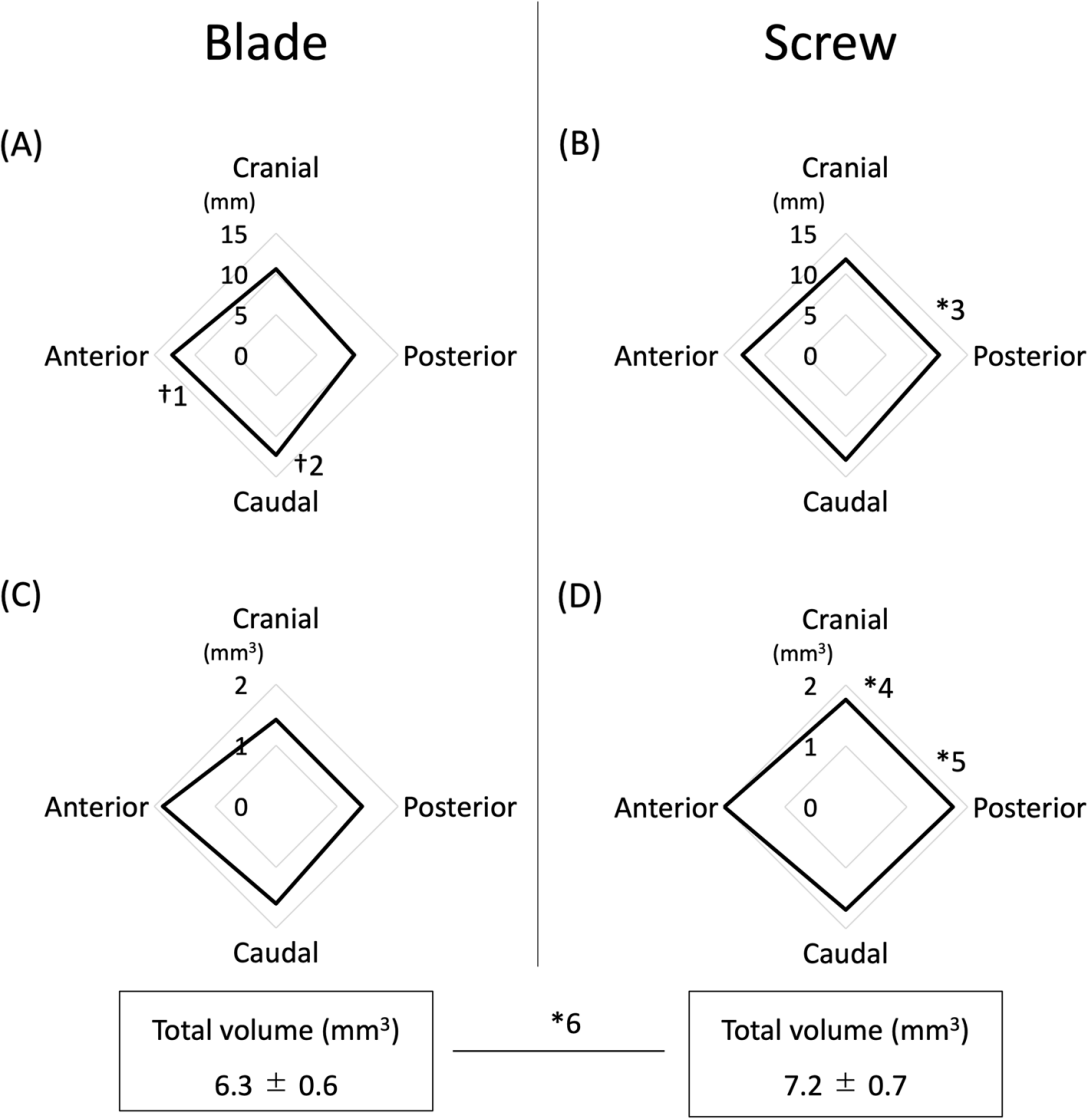
Cement distribution evaluated using CT. The amount of cement injected in four directions was as follows: 1.8 mL in the cranial direction and 0.8 mL each in the other three directions. **A** Maximum penetration depth (MPD) in the Blade group. The anterior (†1) and caudal (†2) MPD was significantly greater than that in the posterior MPD (*p* < 0.01). **B** Maximum penetration depth (MPD) in the Screw group. The posterior MPD (*3) in the Screw group was significantly greater than that in the Blade group (*p* < 0.01). **C** The volume of the head element in the Blade group. Volume in the anterior and caudal direction tended to be larger, but showed no significance. Total volume was 6.3 ± 0.6 mm^3^. **D** The volume of the head element in the Screw group. Volume in the cranial and posterior direction was significantly larger than that in the Blade group (*4; *p* = 0.01, *5; *p* = 0.03). Total volume was 7.2 ± 0.7 mm.^3^, which was significantly larger than that of the Blade group (*6; *p* < 0.01)

Perioperative data are shown in Table 2. Although the intraoperative data were not significantly different between the two groups, one case in the Blade group showed a minute quantity of cement leak into the hip joint through the fracture line. All the head elements of both groups were placed within the center-center position. The Screw group showed a trend towards a larger coronal TAD than the Blade group (10.9 ± 1.8 mm vs. 9.3 ± 2.1 mm; *p* = 0.05). The sagittal TAD in the Screw group tended to be larger than that in the Blade group, but they were not significantly different. As mentioned in the Materials and Methods section, the amount of injected cement in the cranial direction was 1.8 mL, and that in the caudal, anterior, and posterior directions was 0.8 mL each. In the Blade group, MPD in the cranial, caudal, anterior, and posterior directions was 10.6 ± 1.6 mm, 12.3 ± 1.4 mm, 12.8 ± 2.7 mm, and 9.6 ± 1.5 mm, respectively (Fig. 4A). In the Screw group, MPD in the cranial, caudal, anterior, and posterior directions was 11.8 ± 2.1 mm, 12.9 ± 2.1 mm, 12.7 ± 1.8 mm, and 11.5 ± 1.8 mm, respectively (Fig. 4B). In the Blade group, MPD in the anterior and caudal direction was significantly greater than that in the posterior direction (Fig. 4, †1, 2; *p* < 0.01). In the Screw group, MPD in the posterior direction was significantly greater than that in the Blade group (Fig. 4, *3; *p* < 0.01). In the Blade group, volume in the cranial, caudal, anterior, and posterior directions was 1.4 ± 0.3 mm^3^, 1.6 ± 0.3 mm^3^, 1.9 ± 0.6 mm^3^, and 1.4 ± 0.5 mm^3^, respectively. Volume in the anterior and caudal direction tended to be larger, but showed no significance (Fig. 4C). In the Screw group, volume in the cranial, caudal, anterior, and posterior directions was 1.8 ± 0.3 mm^3^, 1.7 ± 0.5 mm^3^, 2.0 ± 0.4 mm^3^, and 1.8 ± 0.3 mm^3^, respectively (Fig. 4D). In the Screw group, volume in the cranial and posterior direction was significantly greater than that in the Blade group (Fig. 4, *4; *p* = 0.01, *5; *p* = 0.03). Total volume in the Screw group was significantly larger than that in the Blade group (7.2 ± 0.7 mm^3^ vs. 6.3 ± 0.6 mm^3^; Fig. 4, *6; *p* < 0.01).

**Table 2:** Perioperative data

	Blade group	Screw group	*p* value
Operative time (minutes)	47.2 ± 22.6	46.3 ± 19.2	0.91
Intraoperative blood loss (mL)	75.5 ± 93.8	36.8 ± 41.3	0.19
Intraoperative complication	1 (cement leak)	None	
Nail length (Short: Long)	11: 3	13: 2	
Reduction quality			
Good	12	11	
Acceptable	2	4	
Poor	0	0	
TAD, coronal (mm)	9.3 ± 2.1	10.9 ± 1.8	0.05
Parker ratio, coronal	0.48 ± 0.03	0.48 ± 0.03	0.85
TAD, sagittal (mm)	9.4 ± 2.1	10.5 ± 1.8	0.15
Parker ratio, sagittal	0.50 ± 0.03	0.49 ± 0.03	0.16
Blade end (mm)	14.2 ± 2.0	13.9 ± 2.9	0.71
Rotation angle (°)	-5.8 ± 10.3	-3.4 ± 12.8	0.60

*TAD* Tip–apex distance

The postoperative evaluation is shown in Table 3. In terms of radiological assessment, there was no significant difference between the two groups in terms of the ΔTAD coronal, ΔTAD sagittal, ΔBlade end, and ΔRotation angle. Similarly, in clinical evaluation, the two groups were not significantly different in terms of ΔParker score, VAS for passive ROM, and VAS for full-load walking. No patients suffered from cut-out / cut through or non-union at final follow-up (the Blade group; 17.1 ± 1.9 months, the Screw group; 16.3 ± 1.3 months). No significant correlations were detected between BMD, T score, YAM, and total cement volume (Table 4).

**Table 3:** Postoperative evaluation

	Blade group	Screw group	*p* value
ΔTAD, coronal (mm)	0.3 ± 0.3	0.3 ± 0.2	0.81
ΔTAD, sagittal (mm)	0.4 ± 0.3	0.2 ± 0.1	0.18
ΔBlade end (mm)	2.6 ± 1.6	2.9 ± 2.0	0.65
ΔRotation angle (°)	0.6 ± 0.3	0.5 ± 0.4	0.61
ΔParker score	− 2.0 ± 1.0	− 2.5 ± 1.3	0.31
VAS for passive ROM	2.6 ± 1.5	2.4 ± 1.1	0.66
VAS for full-load walking	2.4 ± 1.7	2.8 ± 1.8	0.60
Cut-out / Cut-through	0	0	
Non-union	0	0	

*TAD* Tip–apex distance, *VAS* Visual analog scale, *ROM* Range of motion

**Table 4:** Pearson correlation test between DEXA and total cement volume

	Blade group	Screw group
BMD (g/cm^2^)	-0.097	0.101
T score	-0.067	0.193
YAM (%)	-0.095	0.038

*DEXA* Dual-energy X-ray absorptiometry, *BMD* Bone marrow density, *YAM* Young adult mean

## Discussion

The initial fixation of an implant is directly linked to the postoperative pain and subsequent functional recovery. The current randomized study aimed to examine the efficacy of cement augmentation through the TFNA helical blade or lag screw using a detailed CT scan. As previously reported, distribution of 4.2 mL of cement demonstrated that the TFNA helical blade with cement augmentation had double the surface area and triple the volume compared to that without cement augmentation [10]. The current study demonstrated that the position of cement distribution through the lag screw is different from that through the helical blade, and the total volume of the head element is relatively larger in the lag screw. Both groups were similar in terms of clinical outcome, however, such as the mechanical stability after surgery, postoperative pain, and early phase of rehabilitation.

Numerous studies have compared the helical blade to the lag screw without cement augmentation in terms of biomechanics and clinical outcomes. The lag screw needs pre-drilling into the femoral head and results in a loss of useful bone tissue. In contrast, the helical blade is punched in to compress and compact the cancellous bone of the femoral head and to improve the implant stability, especially against rotation in highly osteoporotic bone. In the case of an unstable fracture pattern, including basicervical femoral neck fracture, the surgical procedure of turning the lag screw involves a risk of reduction loss, while driving the helical blade into the femoral head minimizes that risk. One meta-analysis study concluded that cut-through was more common with the helical blades, although cut-out was not significantly different between the helical blades and the lag screws [16]. The other meta-analysis study insisted that the helical blade was demonstrated to require shorter operation and fluoroscopy time; however, outcomes of cut-out, other complications and postoperative function were similar between two groups [17]. Actually, which head element is superior in terms of postoperative implant failure and outcome remains controversial [4, 5, 18, 19].

Although the cause of implant failure is multifactorial, we should avoid inadequate fracture reduction and suboptimal implant position, which depends entirely on operator skill. Weight bearing after surgery promotes the telescoping of the head element and reduces the gap of the intertrochanteric fracture line. If the distal end of the helical blade is buried beneath the lateral wall of the proximal femur or if the set screw for the nail-blade junction is fully locked, the helical blade would be stuck at the inner side of the lateral wall or the blade hole of the nail. In this situation, postoperative transposition at the fracture site might lead to cut-through. Given this, we intend to position the distal end of the helical blade to be slightly protruding from the lateral wall of the proximal femur, and to loosen the set screw by one-quarter turn to allow postoperative telescoping. TAD of the head element is another important factor in preventing implant failure. Although the optimal threshold of TAD (AP + lateral) remains unknown, recent studies have recommended that it be between 20 and 30 mm, because too small TAD can cause cut-through and too large TAD can cause cut-out [6, 19]. Especially in high-risk patients, cement augmentation has been confirmed to be a possible solution to avoid potential implant failure [8, 9].

Resistance force against pushing-in or rotation is essential for preventing complications such as implant failure. Especially in patients with severe osteoporosis, cement augmentation provides sufficient resistance force at the interface between the implant and the cancellous bone. In the coronal plane, the injected cement expanded toward the cranial side where the load is applied and toward the caudal side along the upper edge of the primary compressive trabecula. This corresponds to Ward’s triangle in which the decrease in BMD is significant with aging [20]. Because cement in the cranial direction should be effective against a high weight-bearing load, we injected 1.8 mL of PMMA cement in the cranial direction and 0.8 mL in each of the other three directions. The cranially injected cement tended to flow toward the low BMD site, however. In the Blade group, MPD in the anterior and caudal direction was significantly larger in this study, and we should thus proceed very carefully in these directions and use an image intensifier to detect leakage during cement injection. There was one case of intraoperative cement leak in the Blade group in the current study in which a small crack at the medial calcar was revealed by preoperative CT scan. The cement was not removed as the volume was minimal and did not affect the postoperative rehabilitation. Similarly, Yee et al. reported a single case of cement leakage among their 47 patients [21].

Cement augmentation through the TFNA helical blade doubled the surface area and tripled the volume [10]. In the current study, MPD in the anterior and caudal directions was significantly greater in the Blade group. This is explained by the fact that the region from center to cranio-posterior had higher bone density [22]. Because the cement penetration depth is determined by bone porosity, injection pressure, and trabecular angle orientation, cement might flow towards a region with lower bone density [23]. On the other hand, injected cement in the Screw group was distributed more evenly in the four directions and had a significantly larger total volume than that of the Blade group. There are two possible explanations for this. The first is the difference in surgical procedures between the helical blade and the lag screw. In contrast to the impaction of the cancellous bone in the femoral head during helical blade insertion, the lag screw requires φ11 mm pre-drilling into the femoral head, which results in a loss of dense bone tissue and allows further cement penetration. The second factor was the difference in implant design between the two head elements. The TFNA helical blade has 12 perforated holes, whereas the TFNA lag screw has 8 perforated holes. This difference in the number and position of perforated holes might affect the distribution of cement. All of the cement procedures in this study were performed by the first author. Assuming that cement injection was accomplished within a certain period of time, the small number of perforated holes that act as outlet vents might cause high-pressure injection into the cancellous bone of the femoral head. It remains uncertain which should produce higher mechanical stability: smaller cement volume in the dense bone around the helical blade or larger cement volume in the loose bone around the lag screw. Except for the manufacturer’s information, there has been no research on the biomechanical stability of these two head elements with cement augmentation [24]. In terms of clinical outcome measurement, the two groups were demonstrated to be similar in the current study. As previously reported with a helical blade, cement augmentation with a lag screw also seemed to reduce postoperative pain and accelerate rehabilitation [10, 25].

In severe osteoporotic patients, generally, TFNA with cement augmentation can be evaluated as user-friendly for orthopaedic surgeons who prefer the lag screw over the helical blade. We have some concerns regarding the TFNA lag screw, however. Due to the lateral relief cut of the distal end of the lag screw, the insertion of the lag screw has to be finished every 360 degrees because of set screw fixation from the cranial side. The 360-degree rotation of the lag screw causes 3.5 mm advancement toward the tip of the femoral head, which could be disadvantageous to surgeons aiming for precise TAD. Furthermore, although removal of the TFNA helical blade is easily performed by disrupting the cement pathway at the 12 perforated holes with the pull-out force of hammering, removal of the TFNA lag screw might require counterclockwise rotation torque at the cement–bone interface. Further biomechanical study on rotation torque and back-out force should be conducted in regard to removal of the TFNA lag screw with cement augmentation.

There are several limitations in the present study. First, this was a pilot study with a limited number of patients. Although the previous study about cement distribution had also a relatively small number of patients, further studies with a larger number of patients are needed [9, 10]. Second, the follow-up period in the current study was relatively short. Because the main purpose of this study was to examine the cement distribution and initial fixation, long-term functional evaluation, except for radiological assessment, was missing. Compared to longer follow-up in other studies on cement augmentation, we set a 2-week follow-up time point because clinical outcome within 14 postoperative days is reported to be an important predictive factor reflecting the level of long-term walking ability [26, 27]. Further careful follow-up for hip joint function is necessary, and also the incidence of osteonecrosis of the femoral head should not be overlooked. Third, cement evaluation of the postoperative CT scan was based on only one slice each in coronal and sagittal plane, which might have resulted in erroneous evaluation of MPD. However, because all the head elements were placed in center-center position, the plane crossing axis of the head elements was most important for the initial fixation. Moreover, cement distribution was thoroughly evaluated by the total 36 axial slices (1-mm thickness). Fourth, the impact of the position of the head elements on the cement distribution was not investigated, because all the head elements were placed within the center-center position in the current study.

## Conclusions

The current study investigated the difference in cement distribution between the Blade group and the Screw group. Cement volume through the TFNA lag screw was a size larger than that through the TFNA helical blade. Despite this, both groups had similarly effective results in terms of minimizing implant micro-motion, reducing postoperative pain, and accelerating rehabilitation in the acute phase. In severe osteoporotic patients, the TFNA cement augmentation system is also user-friendly for surgeons who prefer the lag screw over the helical blade.

## Data Availability

The datasets of this study are available from the corresponding author upon reasonable request.

## Abbreviations

TFNA: Trochanteric fixation nail advanced
CT: Computed tomography
MPD: Maximum penetration depth
VAS: Visual analog scale
PMMA: Polymethylmethacrylate
AO/OTA: AO Foundation/Orthopaedic Trauma Association
TAD: Tip-apex distance
AP: Anteroposterior
BMI: Body mass index
ASA: American Society of Anesthesiologists
DEXA: Dual-energy X-ray absorptiometry
BMD: Bone mineral density
YAM: Young adult mean
ROM: Range of motion
